# Supplementary material for: Endemic infectious cutaneous ulcers syndrome in the Oti Region of Ghana: Study of cutaneous leishmaniasis, yaws and Haemophilus ducreyi cutaneous ulcers
Source: PLoS One. 2023 Sep 27;18(9):e0292034. doi: 10.1371/journal.pone.0292034 (PMC10529585; doi:10.1371/journal.pone.0292034)
Supplement: S1 File — (DOCX) [file pone.0292034.s001.docx]

STROBE Statement— Endemic infectious cutaneous ulcers syndrome in the Oti Region of Ghana: study of cutaneous leishmaniasis, yaws and *Haemophilus ducreyi*.

|  | **Item No** | **Recommendation** | **Page No.** | **Relevant text from manuscript** |
| --- | --- | --- | --- | --- |
| **Title and abstract** | 1 | (*a*) Indicate the study’s design with a commonly used term in the title or the abstract | 3 | Line 49; A community based cross-sectional study.. |
|  |  | (*b*) Provide in the abstract an informative and balanced summary of what was done and what was  found | 3 | Lines (48-60); Abstract |
| **Introduction** | | |  |  |
| Background/rationale | 2 | Explain the scientific background and rationale for the investigation being reported | 4-6 | (Lines 78-133) |
| Objectives | 3 | State specific objectives, including any prespecified hypotheses | 6 | Lines (127-133); We therefore hypothesized that yaws, *H. ducreyi* and Buruli ulcer may account for the over 65% of skin ulcers which tested negative for *Leishmania* infection in our previous study. |
| **Methods** | | |  |  |
| Study design | 4 | Present key elements of study design early in the paper | 7 | Lines 150-152; Using a cross-sectional study design, this study was conducted in five communities of the Oti region of Ghana from 11^th^ to 21^st^ September, 2019 |
| Setting | 5 | Describe the setting, locations, and relevant dates, including periods of recruitment, exposure, follow-up, and data collection | 7-8 | Lines 150-167; |
| Participants | 6 | (*a*) *Cohort study*—Give the eligibility criteria, and the sources and methods of selection of participants. Describe methods of follow-up  *Case-control study*—Give the eligibility criteria, and the sources and methods of case | 8. | Methods, Line 165 to 167; Eligible study participants were residents in the study community for > 12 months, aged 2 to 75 years and having atraumatic ulcers (symptomatic for >1 week). |

|  |  | ascertainment and control selection. Give the rationale for the choice of cases and controls  *Cross-sectional study*—Give the eligibility criteria, and the sources and methods of selection of participants |  |  |
| --- | --- | --- | --- | --- |
|  |  | (*b*) *Cohort study*—For matched studies, give matching criteria and number of exposed and unexposed  *Case-control study*—For matched studies, give matching criteria and the number of controls per  case | N/A | This was a cross-sectional study |
| Variables | 7 | Clearly define all outcomes, exposures, predictors, potential confounders, and effect modifiers. Give diagnostic criteria, if applicable | 10 | Lines 197-206 |
| Data sources/ measurement | 8* | For each variable of interest, give sources of data and details of methods of assessment (measurement). Describe comparability of assessment methods if there is more than one group | 10 | Lines 197-212 |
| Bias | 9 | Describe any efforts to address potential sources of bias | 9 | Lines (178 to180); ….As a result, the option was always available for the participants to freely participate in the survey and also, to freely decline to participate in the survey entirely, or in aspects of the survey…. |

|  |  |  |  |  |
| --- | --- | --- | --- | --- |
| Study size | 10 | Explain how the study size was arrived at | 8 | Methods. Line 168 to 171;  All persons with moist atraumatic ulcers in the five CL endemic communities were invited to a central location to participate in the study. The invitation was done through a community-wide announcements information system. |
| Quantitative variables | 11 | Explain how quantitative variables were handled in the analyses. If applicable, describe which groupings were chosen and why | 10 | Methods, Line 207 to 212;  Data were managed using Microsoft Access software version 2013 and analyzed using STATA software version 14. All statistical tested were performed at a 95% confidence level. |
| Statistical methods | 12 | (*a*) Describe all statistical methods, including those used to control for confounding | 10 | Methods, Line 207-212;  Data were managed using Microsoft Access software version 2013 and analyzed using STATA software version 14. All statistical tests were performed at a 95% confidence level. Categorical data were analyzed using the Chi-square test of association and the Fisher’s exact test where cell counts were below 5. The Kruskal Wallis H test was used to assess differences in average ulcer sizes across the ulcer categories. |
|  |  | (*b*) Describe any methods used to examine subgroups and interactions | N/A | There were no subgroups |
|  |  | (*c*) Explain how missing data were addressed | N/A | There was no missing data |
|  |  | (*d*) *Cohort study*—If applicable, explain how loss to follow-up was addressed  *Case-control study*—If applicable, explain how matching of cases and controls was addressed *Cross-sectional study*—If applicable, describe analytical methods taking account of sampling strategy |  | N/A |
|  |  | (*e*) Describe any sensitivity analyses | N/A |  |
| Results |  |  |  |  |
| Participants | 13* | (a) Report numbers of individuals at each stage of study—eg numbers potentially eligible,  examined for eligibility, confirmed eligible, included in the study, completing follow-up, and analysed | N/A | This was a cross-sectional study with only one stage |
|  |  | (b) Give reasons for non-participation at each stage | N/A |  |
|  |  | (c) Consider use of a flow diagram | N/A |  |
| Descriptive data | 14* | (a) Give characteristics of study participants (eg demographic, clinical, social) and information on exposures and potential confounders | 11 | Results, Line 213-227;  Table 1. |

|  |  |  |  |  |
| --- | --- | --- | --- | --- |
|  |  | (b) Indicate number of participants with missing data for each variable of interest | N/A | There was no missing data |
|  |  | (c) *Cohort study*—Summarise follow-up time (eg, average and total amount) | N/A | This was a cross-sectional study |
| Outcome data | 15* | *Cohort study*—Report numbers of outcome events or summary measures over time | N/A | This was a cross-sectional study |
|  |  | *Case-control study—*Report numbers in each exposure category, or summary measures of exposure | N/A | This was a cross-sectional study |
|  |  | *Cross-sectional study—*Report numbers of outcome events or summary measures | 12-15 | Results, Line 229-280 |
| Main results | 16 | (*a*) Give unadjusted estimates and, if applicable, confounder-adjusted estimates and their precision (eg, 95% confidence interval). Make clear which confounders were adjusted for and why they were included | N/A |  |
|  |  | (*b*) Report category boundaries when continuous variables were categorized | 15 | Line 279-280. |
|  |  | (*c*) If relevant, consider translating estimates of relative risk into absolute risk for a meaningful  time period | N/A | There were no estimates of relative risk |
| Other analyses | 17 | Report other analyses done—eg analyses of subgroups and interactions, and sensitivity analyses | N/A | There were no subgroups |
| **Discussion** |  |  |  |  |
| Key results | 18 | Summarise key results with reference to study objectives | 16-18. | Discussion, Line 274-331 |
| Limitations | 19 | Discuss limitations of the study, taking into account sources of potential bias or imprecision.  Discuss both direction and magnitude of any potential bias | 18 | Limitation, Line 283-340 |
| Interpretation | 20 | Give a cautious overall interpretation of results considering objectives, limitations, multiplicity of analyses, results from similar studies, and other relevant evidence | 18 | Conclusion, Line 342 to 350 |

| Generalisability | 21 | Discuss the generalisability (external validity) of the study results | 18 | Conclusion, Line 342 to 350 |
| --- | --- | --- | --- | --- |
| **Other information** |  |  |  |  |
| Funding | 22 | Give the source of funding and the role of the funders for the present study and, if applicable, for the original study on which the present article is based |  | No external funding was received for this study. Collaborators leveraged existing resources at their respective institutions. |
