# Supplementary material for: Endemic infectious cutaneous ulcers syndrome in the Oti Region of Ghana: Study of cutaneous leishmaniasis, yaws and Haemophilus ducreyi cutaneous ulcers
Source: PLoS One. 2023 Sep 27;18(9):e0292034. doi: 10.1371/journal.pone.0292034 (PMC10529585; doi:10.1371/journal.pone.0292034)
Supplement: Attachment — Submitted filename: Response to Reviewers.docx [file pone.0292034.s002.docx]

Epidemiology Department

Noguchi Memorial Institute for Medical Research

P.O Box LG581

University of Ghana

August 9, 2023

The Editor

PLOS NTD

Dear Sir/Madam,

RE: SUBMISSION OF MANUSCRIPT FOR PUBLICATION

Please see below response to comments from academic editor and reviewers in support of the submission of revised manuscript titled “Endemic infectious cutaneous ulcers syndrome in the Oti Region of Ghana: study of cutaneous leishmaniasis, yaws and *Haemophilus ducreyi* cutaneous ulcers” for review and publication.

**Academic editor**

**Comment 1:** Please include a caption for figure 1.

We note that Figures 1 and 3 in your submission contain copyrighted images. All PLOS content is published under the Creative Commons Attribution License (CC BY 4.0), which means that the manuscript, images, and Supporting Information files will be freely available online, and any third party is permitted to access, download, copy, distribute, and use these materials in any way, even commercially, with proper attribution. For more information, see our copyright guidelines: <http://journals.plos.org/plosone/s/licenses-and-copyright>.

We require you to either (1) present written permission from the copyright holder to publish these figures specifically under the CC BY 4.0 license, or (2) remove the figures from your submission:

1. You may seek permission from the original copyright holder of Figures 1 and 3 to publish the content specifically under the CC BY 4.0 license.

We recommend that you contact the original copyright holder with the Content Permission Form (<http://journals.plos.org/plosone/s/file?id=7c09/content-permission-form.pdf>) and the following text:

“I request permission for the open-access journal PLOS ONE to publish XXX under the Creative Commons Attribution License (CCAL) CC BY 4.0 (<http://creativecommons.org/licenses/by/4.0/>). Please be aware that this license allows unrestricted use and distribution, even commercially, by third parties. Please reply and provide explicit written permission to publish XXX under a CC BY license and complete the attached form.”

Please upload the completed Content Permission Form or other proof of granted permissions as an "Other" file with your submission.

In the figure caption of the copyrighted figure, please include the following text: “Reprinted from [ref] under a CC BY license, with permission from [name of publisher], original copyright [original copyright year].

**Response:** The Figure 1 previously included in the manuscript was obtained from a collaborator. However, that Figure 1 has been removed from the manuscript due to non-response from the collaborator for permission to publish the figure.

A new Figure 1 has been included based on results from this study. As corresponding author, I have granted permission for its publication under the Creative Commons Attribution License (CCAL) CC BY 4.0.

Figure 3 was generated from the study being reported and is not based on copyright work of another author. As corresponding author for this submission, I have also granted permission for publication of Figure 3. All other Figures indicated in this manuscript are based on or derived from data obtained from the study being reported. I have therefore granted permission for their publication under the Creative Commons Attribution License (CCAL) CC BY 4.0. I have therefore indicated same in the figure captions.

**Comment 2**: Please review your reference list to ensure that it is complete and correct. If you have cited papers that have been retracted, please include the rationale for doing so in the manuscript text, or remove these references and replace them with relevant current references. Any changes to the reference list should be mentioned in the rebuttal letter that accompanies your revised manuscript. If you need to cite a retracted article, indicate the article’s retracted status in the References list and also include a citation and full reference for the retraction notice.

**Response**: The reference list has been checked. I can confirm that it is complete and correct.

**Feedback from Reviewers:**

Reviewer 2

**Comment:1**- Titles. Long title: The causes of diseases are not written uniformly, often with generic names and sometimes with scientific names.
Suggested short title: Infectious skin ulcer syndrome in Oti region

**Response:** The authors have accepted the proposal for short title and have accordingly amended same at Line 5.

Regarding the causes of diseases not being uniformly written, the authors indicate that their preference was to use the generic names of the skin diseases in the title. In the case of Haemophilus ducreyi cutaneous ulcers however, there is no generic name yet for it beside Haemophilus ducreyi cutaneous ulcer. The authors have therefore changed the text from just ‘Haemophilus ducreyi’ to ‘Haemophilus ducreyi cutaneous ulcer’ to better represent it and to avoid the confusion between Haemophilus ducreyi and Haemophilus ducreyi ulcer (line 3).

**Comment 2**- The lack of uniformity that was suggested about the title also applies to keywords
Comment 3- Please remove the word “Leishmania” from the Key words.

**Response**: The authors accept the suggestion. The word Leishmania has been removed from the key words (line 67).

**Comment 4-** The text of the legend of the tables and images need to be improved

**Response:** The text of the legend of the tables and images have been generally improved.

**Comment 5**- It is not customary to include pictures and tables in the discussion. Eg. L:339

**Response:** The authors agree with the assertion. However, the authors are introducing an algorithm based on a discussion of the results from the study. This explains the introduction of figure 4 at the discussion section.

**Comment 6-** The use of the word microbiome in line 345 is incorrect

**Response**: The authors agree with this comment. The text at line 345 has accordingly been amended.

**Comment 7**- A very long Acknowledgements part

**Response**: The authors accept the comment. Effort has been made to reduce the length of the acknowledgements section (Lines 421-425, 432, 436-440)

**Reviewer #2:**

**Comment 1:** Please explain employed molecular methods, briefly.

**Response**: Molecular methods employed have been better explained (Lines 200-210; 212-221; 225-233)

**Comment 2.** Please provide your molecular results using images.

**Response:** A new figure 1 has been added to present visualization of nested PCR (Ln-PCR) amplification products on 1.5% agarose gel based on tests for *Leishmania* infection.

**Comment 3.** In certain sections of the discussion, like line 289, you merely stated the outcome without providing an accompanying explanation regarding its potential underlying cause.

**Response:** The authors accept the feedback from reviewer. Lines 319 to 324 capture additional explanation provided by authors.

Publishing the findings of this study will significantly impact the current approach and paradigm to diagnosing tropical ulcers in various parts of the world.

Counting on your cooperation.

Yours faithfully,

Richard Adjei Akuffo

(Corresponding author)
